# Characterizing Subjects Exposed to Humidifier Disinfectants Using Computed-Tomography-Based Latent Traits: A Deep Learning Approach

**DOI:** 10.3390/ijerph191911894

**Published:** 2022-09-20

**Authors:** Frank Li, Jiwoong Choi, Xuan Zhang, Prathish K. Rajaraman, Chang-Hyun Lee, Hongseok Ko, Kum-Ju Chae, Eun-Kee Park, Alejandro P. Comellas, Eric A. Hoffman, Ching-Long Lin

**Affiliations:** 1 Roy J. Carver Department of Biomedical Engineering, University of Iowa, Iowa City, IA 52242, USA; 2 IIHR—Hydroscience & Engineering, University of Iowa, Iowa City, IA 52242, USA; 3 Department of Mechanical Engineering, University of Iowa, Iowa City, IA 52242, USA; 4 Department of Internal Medicine, School of Medicine, University of Kansas, Kansas City, KS 66045, USA; 5 Department of Radiology, University of Iowa, Iowa City, IA 52242, USA; 6 Department of Radiology, College of Medicine, Seoul National University, Seoul 100-011, Korea; 7 Department of Radiology, Kangwon National University Hospital, Chuncheon 200-010, Korea; 8 Department of Radiology, Jeonbuk National University Hospital, Jeonju 560-011, Korea; 9 Department of Medical Humanities and Social Medicine, College of Medicine, Kosin University, Busan 600-011, Korea; 10 Department of Internal Medicine, University of Iowa, Iowa City, IA 52242, USA

**Keywords:** humidifier disinfectants, computed tomography, deep learning, cluster analysis, computational fluid and particle dynamics

## Abstract

Around nine million people have been exposed to toxic humidifier disinfectants (HDs) in Korea. HD exposure may lead to HD-associated lung injuries (HDLI). However, many people who have claimed that they experienced HD exposure were not diagnosed with HDLI but still felt discomfort, possibly due to the unknown effects of HD. Therefore, this study examined HD-exposed subjects with normal-appearing lungs, as well as unexposed subjects, in clusters (subgroups) with distinct characteristics, classified by deep-learning-derived computed-tomography (CT)-based tissue pattern latent traits. Among the major clusters, cluster 0 (C0) and cluster 5 (C5) were dominated by HD-exposed and unexposed subjects, respectively. C0 was characterized by features attributable to lung inflammation or fibrosis in contrast with C5. The computational fluid and particle dynamics (CFPD) analysis suggested that the smaller airway sizes observed in the C0 subjects led to greater airway resistance and particle deposition in the airways. Accordingly, women appeared more vulnerable to HD-associated lung abnormalities than men.

## 1. Introduction

Humidifier disinfectants (HDs) are used for the prevention of microorganism growth in humidifiers. In Korea, several types of HD were found to have severe adverse effects on human health. Before they were banned, around nine million people had been exposed to toxic HDs [1]. Many epidemiological studies have demonstrated the relationship of HD with lung injuries and fibrosis [2,3,4,5,6,7,8]. In addition, Kim et al. have demonstrated the aerosolized particles of HD-induced lung inflammatory and fibrotic responses through in vivo and in vitro experiments [9].

Machine learning techniques have been widely used for discovering imaging subtypes of lung diseases with heterogeneous characteristics, such as chronic obstructive pulmonary disease (COPD). For example, Binder et al. used a generative model that captures emphysema subtypes correlated with distinct physiological indicators using an unsupervised approach [10]. In addition, Haghighi et al. employed CT-imaging-based structural and functional variables on both the local and global scales to identify clinically relevant clusters among current and former smokers, respectively [11,12]. However, subjective selections of imaging-based variables or features, which are based on known physiology and pathophysiology, may not be inclusive enough to describe all the features of a heterogeneous lung disease. With the advancement of deep learning techniques, deep learning models are able to detect the important imaging features for the task concerned without human intervention. For example, Li et al. proposed a three-dimensional (3D) convolutional autoencoder with a feature constructor (CAE-FC) that is able to identify imaging-based latent traits which can be used to differentiate phenotypes among COPD patients [13].

HD-associated lung injuries (HDLI) can develop due to multiple causes, and the long-term effects of HD exposure remain unclear. A large portion of people who have claimed that they experienced exposure to HD were not diagnosed with HDLI but still claimed discomfort, possibly due to the influences of HD exposure. Yoon et al. reported the increased risk of asthma symptoms in children with a history of acute bronchiolitis after exposure to HD [14]. In this case, not only exposure to HD, but also the pre-existing bronchiolitis, contributed to the asthma symptoms.

In this study, we aimed to identify subgroups with distinct tissue pattern factors or latent traits derived by computed tomography (CT). The identified subgroups were used to differentiate HD-affected subjects from unaffected subjects among the subjects with normal lung CTs and lung functions. To identify the latent traits, we employed an in-house deep learning model, along with exploratory factor analysis (EFA), to evaluate CT images of both HD-exposed and unexposed subjects [13]. We furthermore applied computational fluid and particle dynamics (CFPD) analysis to elucidate the airway resistance and HD aerosol particle deposition that may characterize HD-exposed subjects with normal-appearing lungs. To our knowledge, this is the first study that uses an unsupervised deep learning approach to discover the subtypes of HD-affected patients, potentially beyond those who can be identified through the manual reading of the CT images. The findings of this study may be useful for the better management of the disease in the future.

## 2. Methods

### 2.1. Human Subject Data and Image Processing

A total of 121 subjects were selected for investigation. We retrospectively collected inspiratory (IN) and expiratory (EX) CT image data acquired at total lung capacity (TLC) and residual volume (RV), as well as PFT results. Among these, 96 subjects claimed exposure to toxic HD but had normal CT and pulmonary function test (PFT) results. The most common HD chemical ingredient was polyhexamethylene guanidine (PHMG). Other major ingredients were oligo (2-)ethoxyethoxyethyl guanidine chloride (PGH), chloromethylisothiazolinone (CMIT), and methylisothiazolinone (MIT) [15]. The exposure type and duration were defined based upon the questionnaire. The other 25 subjects did not claim exposure to toxic HD and had normal-appearing CT and PFT results. The demographic data and PFT measurements for each stratum are shown in Table 1. The study protocol was approved by the institutional review boards (IRB) of Seoul National University Hospital (SNUH) and Jeonbuk National University Hospital (JBNUH) (project title: Quantitative CT features of potential respiratory abnormalities in children and adults with normal-appearing chest CT by humidifier disinfectant inhalation exposure). For the current research, informed consent was waived by IRB at SNUH, because this was a retrospective study based upon existing data and the research involved minimal risks. The research collected only non-identifiable data. All methods were carried out in accordance with the relevant institutional guidelines and regulations.

Patients underwent 128 multidetector CT scanning at full inspiration and full expiration using a Philips Ingenuity scanner (Philips Healthcare, Cleveland, OH, USA) in SNUH and Somatom Definition Flash scanner (Siemens Healthcare, Forchheim, Germany) in JBNUH. The CT parameters were as follows: tube voltage (120 kVp), tube current (SNUH: inspiration, DRI (dose right index) 14, expiration, DRI 14; JBNUH: inspiration, 110 quality reference mAs, expiration, 50), slice thickness (1.0 mm), reconstruction interval (1.0 mm), reconstruction algorithm (SNUH: standard (B); JBNUH: B35f), and rotation time (0.5 s).

**Figure 1:**
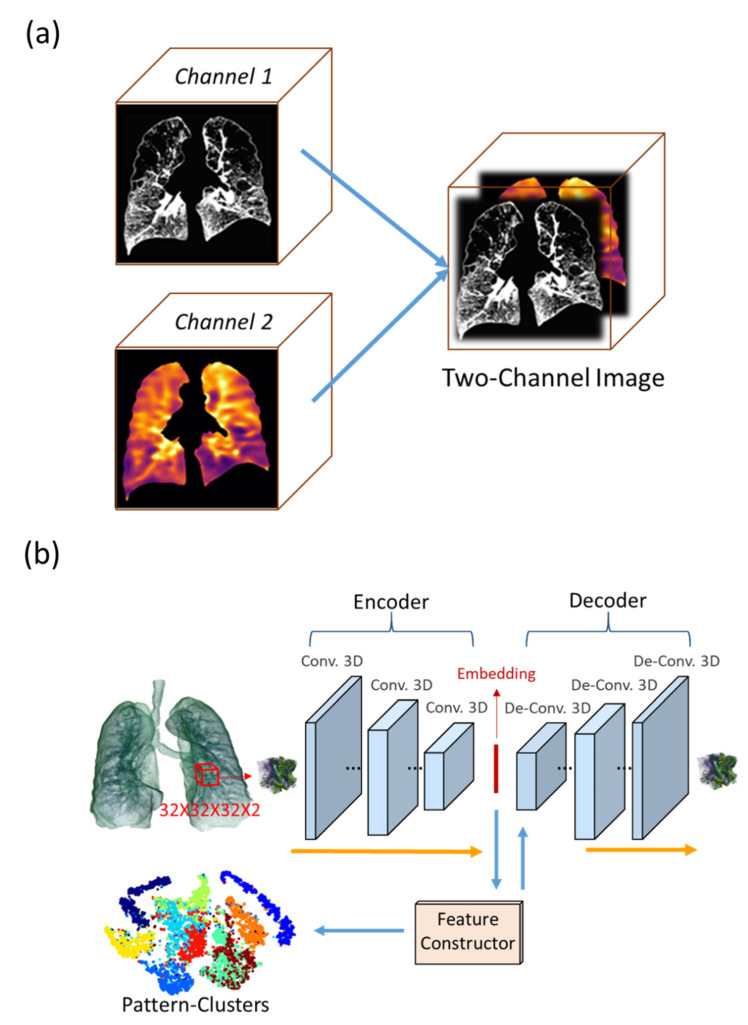
(**a**) A two-channeled image was constructed using the IN image as the first channel and the Jacobian mask as the second channel. (**b**) Schematic of the CAE-FC model. Reprinted/adapted with permission from Ref. [13], 2021, Li et al. The ROIs were fed into the 3D CAE-FC model to obtain their one-dimensional (1D) representations and to group the ROIs with similar patterns into pattern clusters.

The determinant of the Jacobian matrix (*J*) was computed by registering IN and EX images [16,17] for each voxel in the IN images so as to measure local volume change. In this study, *J* is defined as the EX to IN volume ratio; thus, 1/*J* indicates the local volume expansion. Two-channeled images were constructed by combining the *J* values with the IN images (Figure 1a). In total, 98,250 3D regions of interest (ROIs) were randomly extracted from the two-channeled images. The size of the ROI was approximately the size of a secondary pulmonary lobule (20 × 20 × 20 mm^3^), which is a fundamental unit of the lung structure [18]. We used Apollo software (VIDA Diagnostics, Coralville, Iowa) for the segmentation of the lungs and airways and Insight Toolkit (ITK, version 5.0, Kitware, Clifton Park, NY, USA) and in-house software for the further image processing. The detailed information about the image processing can be found in Li et al. [13].

### 2.2. 3D Convolutional Autoencoder (CAE) and Feature Constructor (FC)

Each voxel in the extracted ROIs consisted of two input data channels: rescaled CT density and *J*. The ROIs were fed into a 3D CAE-FC model to obtain their one-dimensional (1D) representations and to group the ROIs with similar patterns into pattern clusters [13]. The optimal number of pattern clusters was determined by testing different numbers of pattern clusters and selecting the number which yielded the lowest loss during the training of the CAE-FC model (Figure S1).

The CAE-FC model contained an encoder, which consisted of 3D convolutional layers, an embedding layer, and a decoder, which consisted of 3D de-convolutional layers and a FC (Figure 1). The FC suppressed the small activations in the embeddings and forced the decoder to reconstruct the ROIs using only the important activations. The ROIs were grouped based on the greatest activations among the highest-level feature maps (embeddings). Therefore, the CAE-FC model was able not only to extract the 1D representations of the ROIs but also to group the 1D representations with similar patterns. Please refer to [13] for more detailed information about the CAE-FC model.

**Figure 2:**
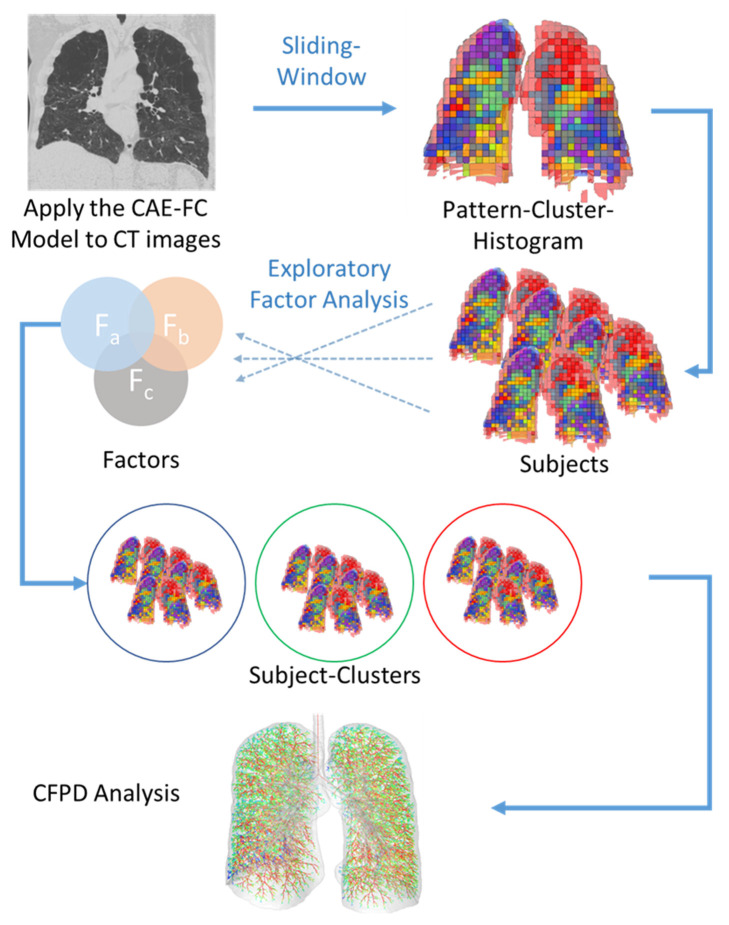
The CAE-FC model is applied to the ROIs extracted by the sliding window technique so as to classify the lung tissue patterns and construct a pattern cluster histogram for each subject. EFA is then used to extract the latent traits (factors) from the pattern cluster histograms of the subjects. Using the factor scores, subject clusters are identified by the k-means clustering technique. Finally, CFPD analysis is conducted to evaluate the particle depositions of the representative subjects.

After the CAE-FC training, a sliding window technique was applied to classify the lung tissue patterns and quantify the frequency of each pattern cluster within the whole lung. Then, a pattern cluster frequency histogram could be calculated for each subject. The procedure is illustrated in Figure 2. We further used EFA [19,20,21], a data reduction technique, to extract latent traits (factors) from these pattern clusters. That is, highly associated pattern clusters were grouped together into the same factor, and the pattern cluster frequencies of the subjects were then transformed into factor scores. Please refer to the Supplementary Materials for the steps of the EFA.

### 2.3. Factor Interpretation

The averaged intensities and the averaged *J* of the pattern clusters showing high associations with each factor were compared. In addition, to facilitate the interpretation of the identified factors, we evaluated the correlations of the factors with the clinical and CT imaging-based variables. The included clinical variables were age, gender, height, weight, body mass index (BMI), percent-predicted forced expiratory volume in 1 s (FEV_1_%), and forced vital capacity (FVC). The included imaging-based variables were the low attenuation area (LAA) percentage at RV (LAA_RV_%) in the total lung and in each lobe, tissue fraction at TLC (Tissue%) in the total lung and in each lobe, LAA percentage at TLC (LAA_TLC_%) in the total lung and in each lobe, functional small airway disease percentage (fSAD%) in the total lung and in each lobe, airway tree to lung volume ratio (AWV%) [22,23], and CT-measured RV to TLC ratio (RV/TLC) [24].

### 2.4. Identification of the Subject Clusters

K-means clustering was applied to identify the subject clusters within the factor scores. The number of subject clusters was determined by evaluating the inter-cluster and intra-cluster variability. Next, the correlation between the clusters and exposure was tested. Moreover, the inter-cluster differences in terms of the aforementioned clinical and imaging-based variables were examined. Lastly, a decision tree model, viz., a non-linear feature selector, was trained and developed using both the clinical and imaging-based variables, showing significant differences between clusters. Thus, this model may potentially be used in clinical practice to assist in the identification of HD-associated lung abnormalities in susceptible patients.

### 2.5. Simulations of Airflow in the Airways Using CFPD

To facilitate the interpretation of the subject clusters in terms of the inhaled HD aerosol deposition, a HD-exposed subject and an unexposed subject were selected for CFPD analysis, who were chosen from the clusters with the minimal and the largest proportions of HD-unexposed subjects, respectively. The selection was based on their proximity to the clusters’ geometric centers in the feature space of the factor scores. Moreover, the mean hydraulic diameters of the lobar and sub-lobar subsets were calculated for the two clusters in order to facilitate the explanation of the CFPD results. The lobar airways were defined as the left upper lobe (LUL) bronchus, left lower lobe (LLL) bronchus, the right upper lobe (RUL) bronchus, right middle lobe (RML) bronchus, and right lower lobe (RLL) bronchus. In this study, the sub-lobar subset airways were defined as the child and grandchild branches of the lobar branches.

Subject-specific flow rates were applied at the supraglottal inlet. The inhalation flow pattern was approximated by a sinusoidal waveform. The inhaled air volume was set to a tidal volume, which was estimated using 7 mL of air per kilogram of body weight [25]. For all cases, the time to peak inspiration was set to 1.2 s with an inhalation period (T) of 4.8 s. An in-house three-dimensional (3D) CFPD lung model was used to simulate the velocity field and pressure drop in the central airways, which can be used to calculate the airway resistance [26,27]. Subsequently, the trajectories of 0.5 µm spherical particles, which were used to model the HD particles, were computed using a Lagrangian particle-tracking algorithm [27] in order to capture the local hotspots of high deposition density on the walls of the 3D airways. We further applied a 1D deposition model to predict the deposition fractions in the entire subject-specific airway tree [28,29,30]. Particle depositions in the entire conducting airway were estimated for the representative subjects during inspiration.

### 2.6. Statistical Analysis

The differences between the means of the independent groups were analyzed by Welch’s ANOVA with the Games–Howell method for post hoc pairwise tests. Pearson’s correlation and bi-serial correlation were used to examine the associations of the identified factors with the known continuous variables and binary variables, respectively. The chi-square test was used to examine the relationship between two categorical variables. The significance level was set as 0.05. The statistical analyses were conducted using SciPy 1.4.1 and Pingouin 0.3.4 in the Python 3 packages.

## 3. Results

**Figure 3:**
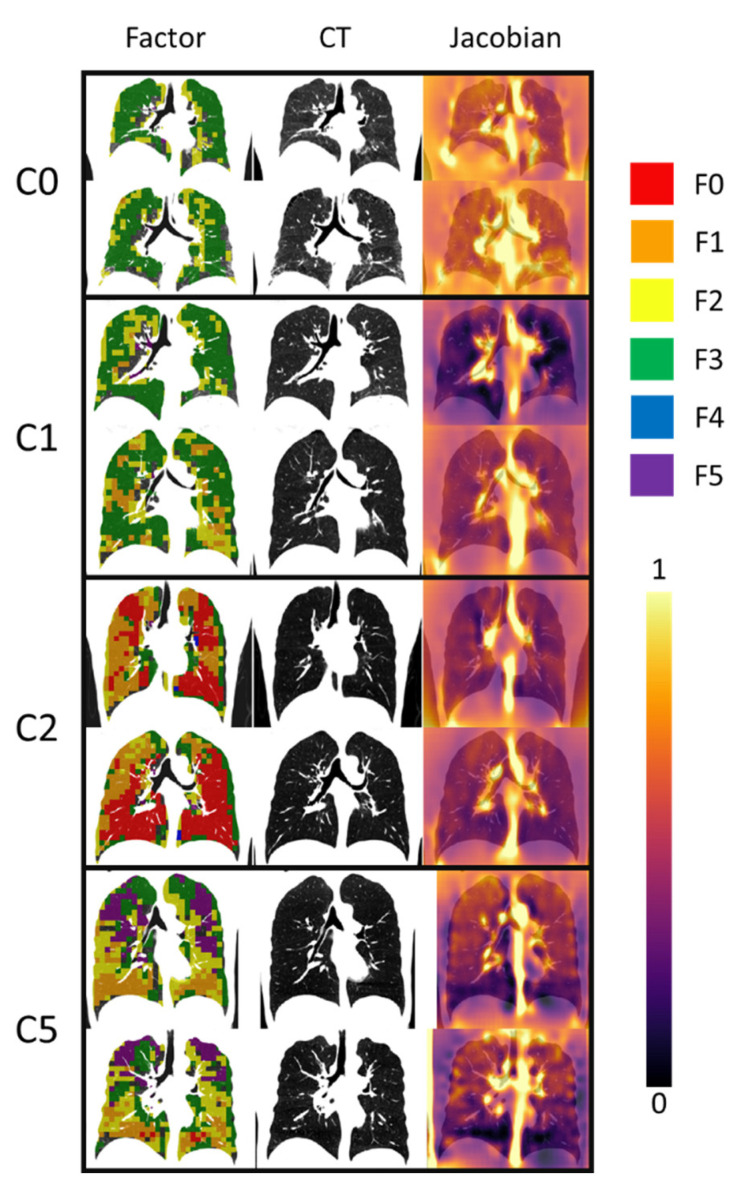
Comparison of two representative subjects each for C0, C1, C2, and C5. Factor masks (**left** column) and Jacobian masks (**right** column) were overlapped with CT images (**middle** column).

The deep learning and the following statistical analyses were conducted for the 121 subjects. Among them, 96 subjects (79.3%) claimed exposure to HD and 25 subjects (20.7%) did not. A total of 80 pattern clusters were identified by the CAE-FC model, and the quantification of the pattern clusters within the lungs was performed to generate the pattern cluster histograms. Subsequently, six factors (F0–F5) were extracted from the pattern cluster histograms of all subjects. Finally, six clusters (C0–C5) were obtained by k-means clustering in the feature space of the factor scores. The demographic data, PFT measurements, and exposure information for each cluster are shown in Table 2. The exposure frequency for each cluster is shown in Table 3. A significant relationship (*p* < 0.001) between exposure to HD and the clusters showed that the frequencies of HD-exposed subjects in the clusters were different from one another. C0 had the smallest fraction (3.1%) of HD-unexposed subjects, while C5 had the largest fraction (48.5%) of HD-unexposed subjects. On the other hand, the proportions of HD-unexposed subjects in C1 (13.0%) and C2 (17.9%) were similar to that of the entire dataset, indicating that exposure to HD was not the main feature differentiating C1 and C2 from the entire dataset. C3 and C4 were excluded from the analysis, since the number of members was small. Thus, hereafter, we presented the results for the four main clusters of C0, C1, C2, and C5, with a focus on C0 and C5, to characterize the HD-exposed subjects, regarding C5 as cluster of unexposed subjects with no HD-induced abnormal features. We note that the majority (64.0%) of unexposed subjects were found in C5. Nevertheless, neither the exposure type nor the exposure duration were associated with the cluster membership (Table 2). As an illustration, Figure 3 shows a comparison of the factors, CT densities, and *J* values of the representative subjects for the four clusters.

**Figure 4:**
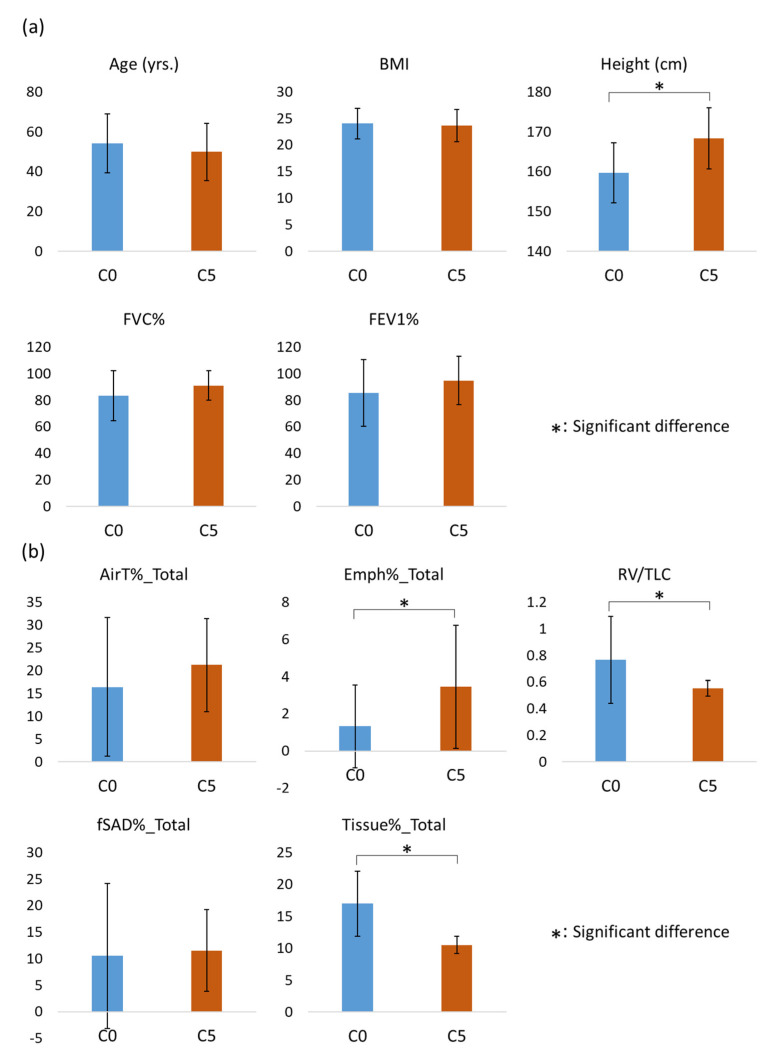
(**a**) Comparison between the clinical variables of C0 and C5. (**b**) Comparison between the imaging-based variables measured at total lung capacity of C0 and C5. The differences between the means of independent groups were analyzed by Welch’s ANOVA with the Games–Howell method for post hoc pairwise tests and the significance level was set as 0.05.

**Figure 5:**
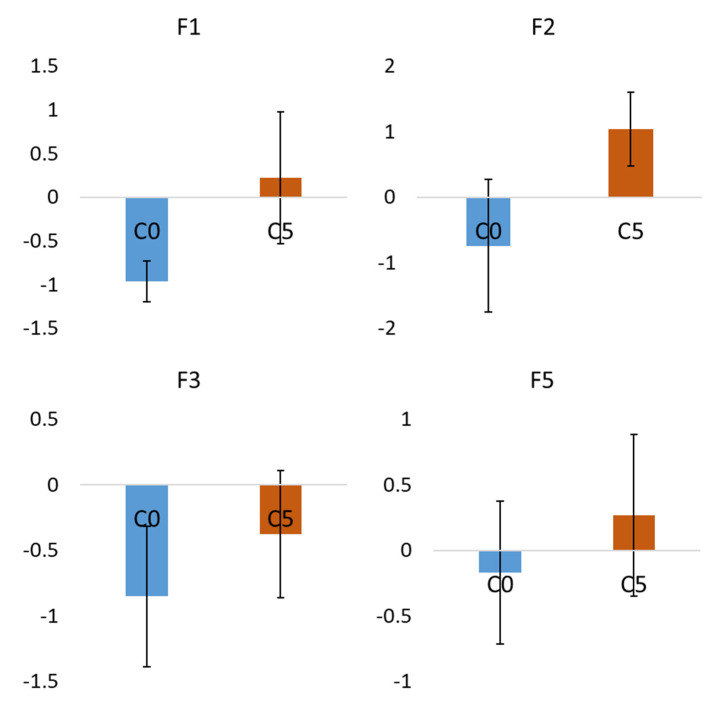
Mean factor scores which account for the differences between C0 and C5. Significant differences between C0 and C5 were found for F1, F2, F3, and F5. The differences between the means of independent groups were analyzed by Welch’s ANOVA with the Games–Howell method for post hoc pairwise tests and the significance level was set as 0.05.

A significant relationship (*p* < 0.001) between gender and the clusters was found. C0 was female dominated (59.4%), while C5 was male dominated (84.8%). On the contrary, there was no significant difference between the means of the clusters in terms of FVC% and FEV_1_%. The pairwise comparisons showed that C0 was significantly smaller than C5 in terms of height (C0: 159.74 ± 7.52 cm; C5: 168.33 ± 7.61 cm) and LAA_TLC_%_Total (C0: 1.33 ± 2.23%; C5: 3.46 ± 3.30%). On the other hand, C0 was significantly larger than C5 in terms of RV/TLC (C0: 0.76 ± 0.33; C5: 0.55 ± 0.06) and Tissue%_Total (C0: 16.98 ± 5.07%; C5: 10.51 ± 1.36%). However, there were no significant differences between C0 and C5 in terms of LAA_RV_%_Total (C0: 16.43 ± 15.22%; C5: 21.21 ± 10.24%) and fSAD%_Total (C0: 10.52 ± 13.66%; C5: 11.53 ± 7.69%). The mean values and standard deviations of these variables for each cluster are shown in Figure 4. Significant differences were found between C0 and C5 for F1, F2, F3, and F5 (Figure 5). Among these factors, F2 and F3 were found to be bi-polar factors. A bi-polar factor is a latent trait that has two opposite directions. That is, their positive and negative factor scores were contributed by pattern clusters with positive loadings and negative loadings, respectively. For example, when describing the air content in a lung, both low attenuation and high attenuation areas of the CT image can be used. That is, low and high attenuation areas correspond to more air content and less air content in a lung, respectively.

**Figure 6:**
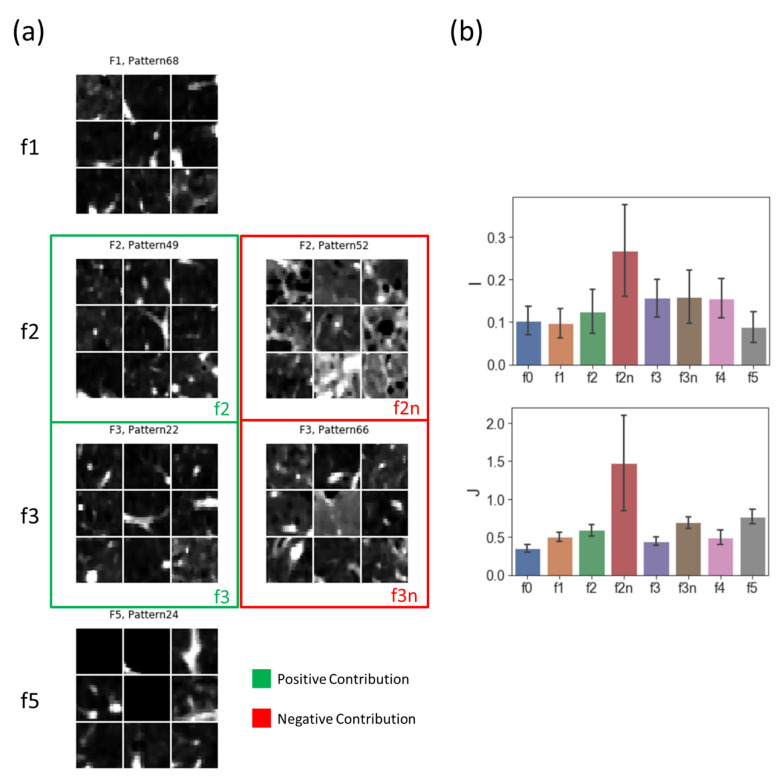
(**a**) Samples of the pattern clusters that showed a high contribution to the factors of F1, F2, F3, and F5. These four factors can differentiate C0 from C5. F2 and F3 are bi-polar factors; thus, there are pattern clusters with either positive (f2 and f3 in green boxes) or negative (f2n and f3n in red boxes) contributions to them. (**b**) Averaged CT attenuation (I) and averaged Jacobian (J) of the pattern clusters which had showed a contribution to each factor.

The mean CT density and *J* of the pattern clusters were calculated. The pattern clusters that were highly positively associated with each factor were denoted as f0−f5. The pattern clusters that were highly negatively associated with F2 and F3 were denoted as f2n and f3n, respectively (i.e., pattern clusters associated with the negative portion of the bi-polar factors). We found that f1 and f2 had a relatively low CT density and *J* (f1: CT density = 0.097, *J* = 0.504; f2: CT density = 0.125, *J* = 0.588), f3 had a high density but low *J* (CT density = 0.156, *J* = 0.442), and f5 had a low CT density but high *J* (CT density = 0.088, *J* = 0.769). On the other hand, f2n had both the highest CT density and *J* (CT density = 0.267, *J* = 1.470), and f3n had a relatively high CT density and *J* (CT density = 0.159, *J* = 0.690, Figure 6b). Examples of f1, f2(n), f3(n), and f5 are shown in Figure 6a.

**Figure 7:**
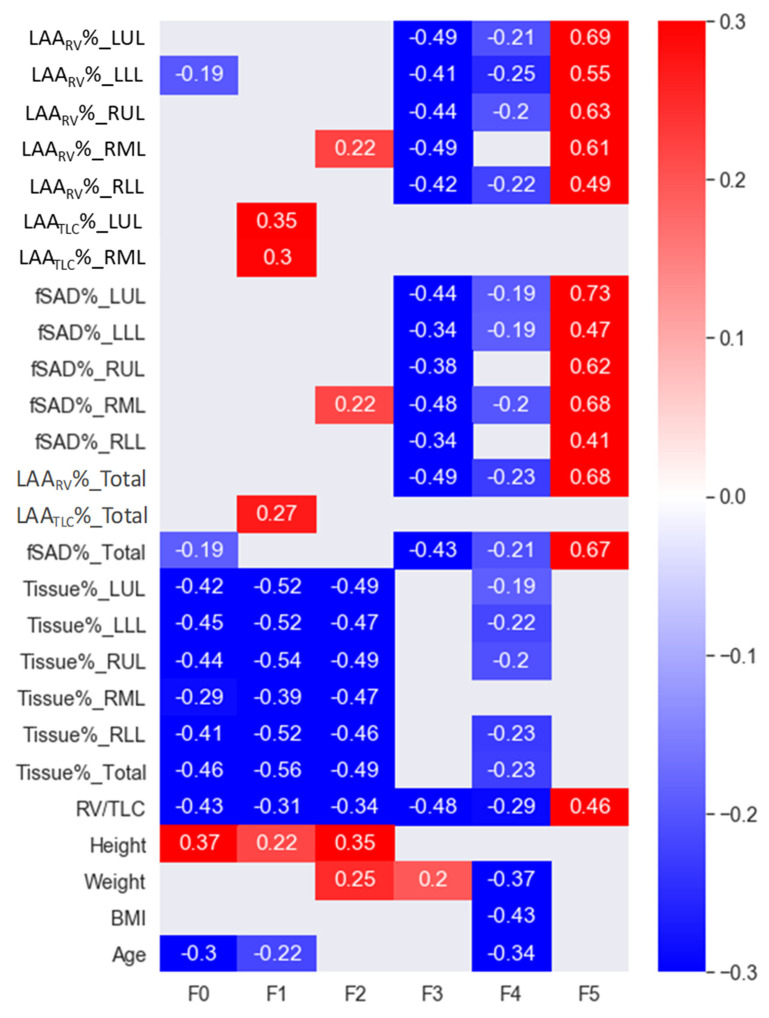
Pearson’s correlations between factors and common clinical data and imaging-based variables. Only correlations with *p* values of less than 0.05 are shown in the figure.

Correlations between the factors and common clinical and imaging-based variables were also calculated in order to better interpret the results (Figure 7). F1 and F2 were negatively correlated with Tissue%_Total and Tissue% for all the lobes. F3 was negatively correlated with LAA_RV_%_Total and LAA_RV_% for all the lobes, while F5 was positively correlated with LAA_RV_%_Total and LAA_RV_% for all the lobes. F1, F2, and F3 were negatively correlated with RV/TLC. On the contrary, F5 was positively correlated with RV/TLC. Moreover, F1 was positively correlated with LAA_TLC_%_Total, LAA_TLC_%_LUL, and LAA_TLC_%_RML, while F5 was positively correlated with fSAD%_Total and fSAD% for all the lobes. However, F1, F2, F3, and F5 were not strongly correlated with the clinical variables of BMI, weight, height, age, FEV_1_%, and FVC%.

**Figure 8:**
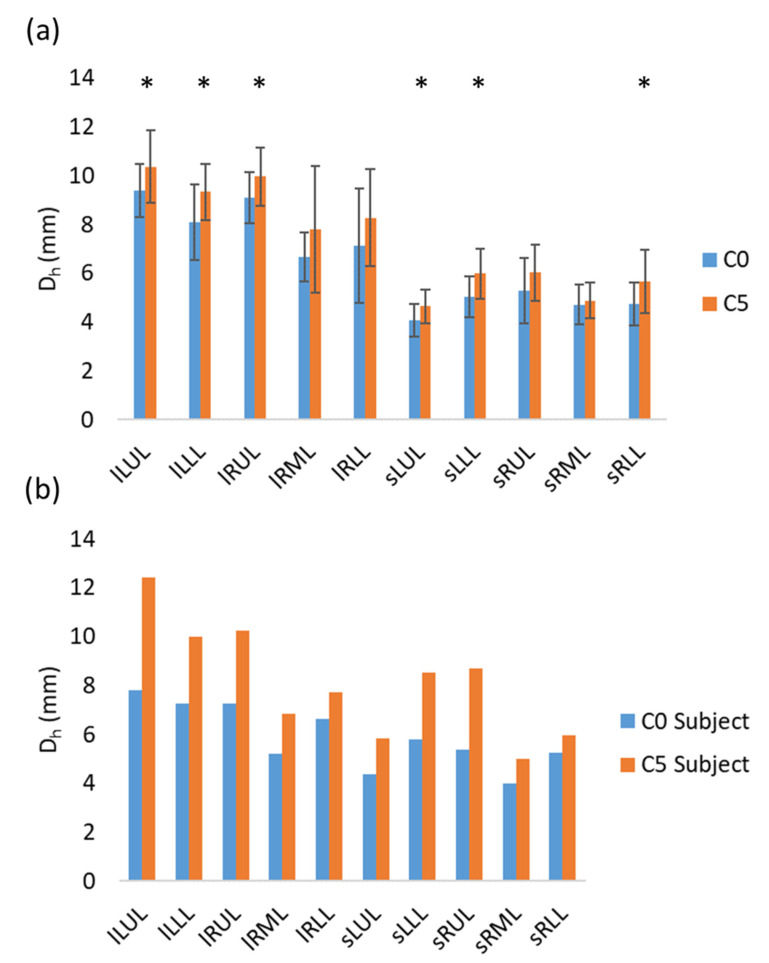
(**a**) Averaged hydraulic diameters of the lobar airways and segmental airways of C0 and C5. A “*” denotes a significant difference between C0 and C5. The differences between the means of independent groups were analyzed by Welch’s ANOVA with the Games–Howell method for post hoc pairwise tests and the significance level was set as 0.05. (**b**) Hydraulic diameters of the lobar airways and sub-lobar subset airways of the C0 and C5 representative subjects. A lobar airway and a sub-lobar subset airway in a lobe are denoted as “l” followed by the abbreviation of the given lobe (e.g., lRUL) and “s” followed by the abbreviation of the given lobe (e.g., sRUL), respectively.

**Figure 9:**
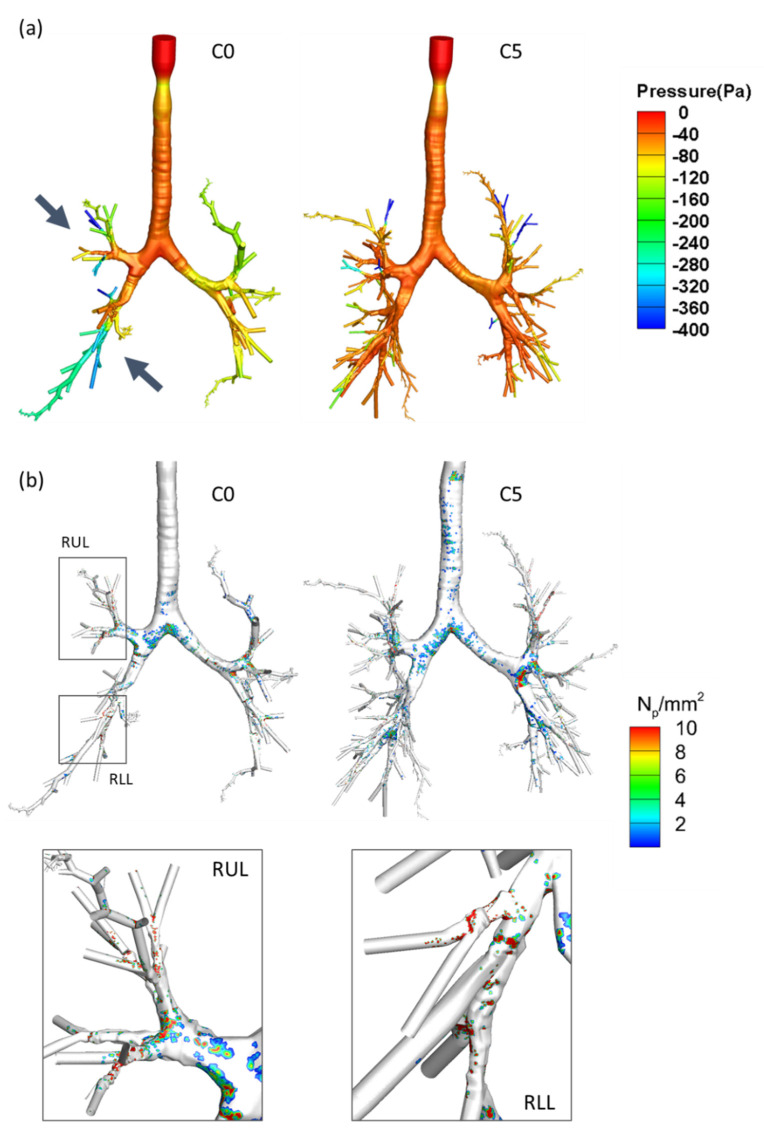
(**a**) Peak inspiratory pressure distributions in the airways. The arrows represent the greater pressure drop in the C0 representative subject. (**b**) Color maps of the particle deposition density on the airway wall surface (N_p_/mm^2^). Hotspots were found in the segmental airways of RUL and RLL in the C0 representative subject.

**Figure 10:**
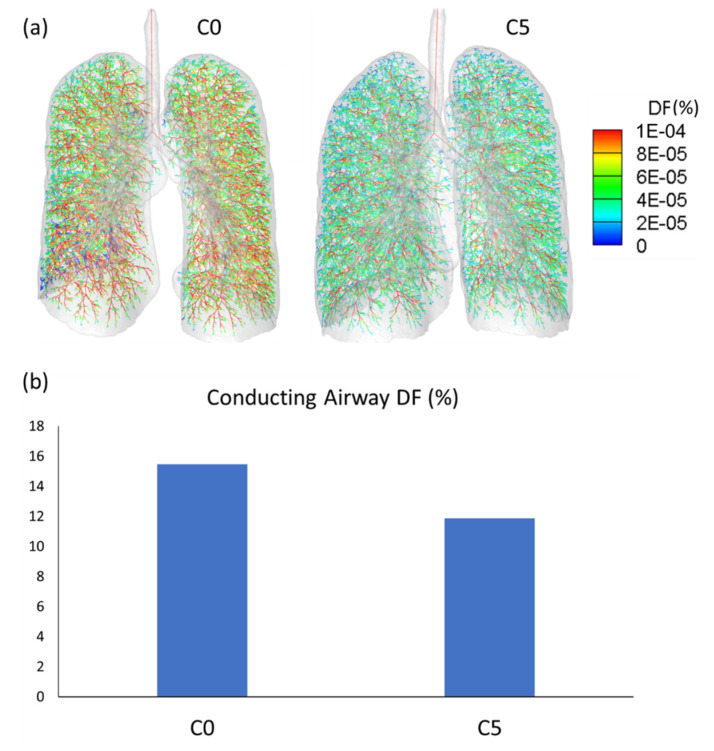
(**a**) Deposition fractions (DF) at each individual airway branch of the entire conducting airway estimated by the 1D particle deposition simulation of the C0 and C5 representative subjects. (**b**) The total lung deposition fraction in the conducting airways of the C0 and C5 representative subjects during inspiration. The C0 representative subject demonstrated a greater particle deposition in the conducting airways.

The lobar and segmental airways of C0 subjects were found to have smaller hydraulic diameters than those of C5 subjects, although no significant difference was found between the lobar airways in RML and RLL or the segmental airways in RUL and RML (Figure 8a). Similarly, the hydraulic diameters of both lobar and segmental airways were found to be smaller in the C0 representative subject (Figure 8b). The CFPD analysis showed that the C0 representative subject had a greater pressure drop in both the lobar and segmental airways of all lobes except for RML (Figure 9a). Hotspots were observed in the segmental airways of RUL and RLL in the C0 representative subject (Figure 9b). Moreover, a larger amount of particle depositions in the conducting airway were found in the C0 representative subject compared to the C5 representative subject (Figure 10). These results suggest that the C0 subjects experienced airway narrowing, which induced increased airway resistance and greater particle deposition.

**Figure 11:**
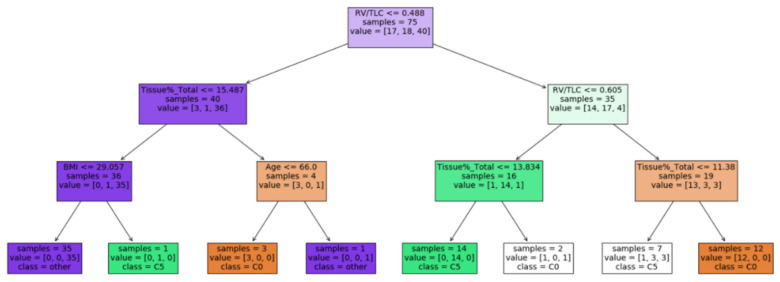
A decision tree model which takes the imaging-based variables and the clinical variables as inputs and predicts the cluster membership.

A decision tree was trained using the selected inspiratory CT imaging metrics and clinical variables for the classification of the subjects into either C0, C5, or other clusters (Figure 11). An accuracy of 0.79 was achieved based on a three-fold cross-validation analysis.

## 4. Discussion

In this study, we extracted six factors from lung tissue patterns. Subsequently, the factors were used to identify four major clusters of C0, C1, C2, and C5 from a total of 121 either HD-exposed or unexposed subjects with normal-appearing lungs. Table 4 summarizes the characteristics of each cluster. The association between the clusters and HD exposure indicated that the clusters differentiate HD-exposed from unexposed subjects. Among the clusters, C0 had the largest fraction (96.9%) of HD-exposed subjects, while C5 had the smallest fraction (51.5%) of HD-exposed subjects (Table 3). The majority of unexposed subjects (16 of 25, 64.0%) were in C5, which suggests that C5 does not reflect HD-specific abnormalities. Thus, HD-exposed subjects in C5 may be regarded as HD-unaffected subjects, although they claimed exposure to toxic HD. In contrast, subjects in C0, as well as C1 and C2, may reflect HD-affected abnormality and could be considered as candidates for a follow-up study on progression or recovery. F1, F2, F3, and F5 are the major features that differentiate C0 and C5.

C0 was dominated by HD-exposed female subjects. It was characterized by negative scores of F2 and F3, suggesting high CT attenuation, high *J* (low expansion), increased Tissue%, and increased RV/TLC. Moreover, f2n appears to be associated with regional hypo-inflation and subpleural opacities (Figure 6), while f3n may be associated with air trapping or low attenuation on expiratory CT. The effects of f2n and f3n may lead to opposite results in regard to lung attenuation. In C0, LAA_RV_% and fSAD% were smaller than those of C5, which implies that the influence of f2n was stronger than that of f3n in C0. Increased Tissue% may reflect an increased high attenuation area in an inspiratory image, which is attributable to inflammation or fibrosis. These findings were obtained by the presence of mosaic attenuation and air trapping. In addition, an increase in RV/TLC was due to decreased TLC, that is, hypo-inflation, implying a fibrotic lung [31]. Mosaic attenuation and air-trapping observed from CT images, as well as decreased TLC, are features similar to those of fibrotic hypersensitivity pneumonitis [31,32]. It has been suggested that HD can damage epithelial cells and induce inflammation, leading to excessive tissue repair and lung fibrosis [3,9].

C5 was dominated by unexposed male subjects. Regarding the identified factors, this cluster was characterized by positive scores of F2 and F5, demonstrating relatively greater LAA_RV_%, LAA_TLC_%, and fSAD% than the HD-exposed-subject-dominated clusters. While C5 was dominated by subjects from the control group, it is noteworthy that C5 subjects were greater in height. For the HD-exposed subjects in C5, we could assume they were unaffected by HDs or in the resolving stage, by which point CT features have diminished into faint lesions or disappeared [33].

C1 and C2 were also predominated by HD-exposed subjects (Table 3). While the characteristics of C1 and C2 are still unclear and further investigation is needed, we provided a brief discussion. C1 and C2 were associated with greater F3 and F1, respectively. F3 and F1 both showed greater lung deformation (smaller *J*, Figure 6b). This might be contributed by hyper-deflation of the lung in RV (Table 4). The TLC volume is not significantly different from C5. More information for C1 and C2 can be found in Table S1.

From the CFPD analysis, we found an increase in the pressure drop in both the lobar and segmental airways of all lobes except RML in the C0 subject. In addition, particle deposition hotspots were observed in the segmental airways in RUL and RLL of the C0 representative subject. Similarly, more particle depositions were found in the complete conducting airways of the C0 representative subject. Furthermore, the increase in the pressure drop might be attributed to a decrease in the hydraulic diameters of the lobar and segmental airways. Therefore, we speculated that females are more susceptible to HD-associated lung abnormality because of their smaller airway size, which may lead to greater airway resistance and more HD particle accumulation in small airways.

However, there may exist other environmental or occupational factors that can also contribute to the inclination of females to be more vulnerable to HD-associated lung abnormality. For example, housewives tend to spend longer periods of time at home, resulting in more exposure to HD. It has been reported that females and children were at greater risk with a longer duration of exposure [2,34]. This study has several limitations. First, this is a retrospective study. The duration and dose of exposure to HDs were not well controlled. The data from the questionnaire related to HD exposure were collected from the subject years after the HD exposure. Therefore, there is strong possibility of exposure misclassification due to erroneous statements based on recall bias and other technical reasons [33]. Moreover, there are many types of HD. Each type may have different effects on the patients. Thus, these factors may affect the interpretation of the findings.

The decision tree model (Figure 11) used the lung deformation (RV/TLC) and tissue content in the lung (Tissue%) to differentiate the clusters. This coincides with the previous result, showing that C0 subjects had less lung deformation and a greater tissue content than the other clusters, implying more fibrotic or inflammatory lungs in the C0 subjects. Nevertheless, the derivation of RV/TLC and Tissue% required the image processing of CT images. The decision tree model could serve as a diagnostic aid for clinical doctors.

## 5. Conclusions

Among those subjects with normal-appearing lung CT and normal PFT results, HD-affected (C0) and HD-unaffected (C5) subject clusters were identified by CT-based two-channel deep learning latent traits. C0 was characterized by increased Tissue% and hypo-inflation, as compared to C5, suggesting that our model can distinguish HD-associated lung abnormalities from among a normal-appearing HD-exposed population, and that C0 subjects may be at risk of HD-induced inflammation or lung fibrosis. The CFPD analysis suggested that HD-affected subjects tend to have a higher airway resistance and higher HD aerosol particle deposition density, which may increase their susceptibility to HD-induced lung damage. Finally, we developed a decision tree model to assist in the identification of HD-associated lung abnormalities in susceptible subjects. The findings of this study may be useful for the future studies aiming to follow up HD-exposed normal-appearing subjects. 

## Figures and Tables

**Table 1:** Demographic data and PFT measurements for HD-exposed and unexposed subjects. Entries are mean (SD) unless otherwise specified. The differences between the means of independent groups were analyzed by Welch’s *t*-test and the significance level was set as 0.05. Chi-square test was used to examine the relationship between two categorical variables.

	Exposed (*n* = 96)	Non-Exposed (*n* = 25)	Total (*n* = 121)	*p*
**Age (yrs.)**	49.86	45.44	48.95	0.116
	(15.43)	(11.38)	(14.75)	
**BMI (kg/cm^2^)**	23.40	24.72	23.67	0.057
	(3.21)	(2.94)	(3.19)	
**Height (cm)**	163.95	169.96	165.20	0.002
	(7.89)	(8.15)	(8.28)	
**Weight (kg)**	63.11	71.76	64.91	0.003
	(10.94)	(12.06)	(11.68)	
**FVC (% of pred)**	87.16	99.28	89.68	<0.001
	(16.64)	(7.17)	(15.92)	
**FEV_1_ (% of pred)**	89.53	107.72	93.32	<0.001
	(20.86)	(10.51)	(20.52)	
**Gender (%)**	45.8/54.2	16.0/84.0	39.7/60.3	0.013
**(Female/Male)**				

**Table 2:** Demographic data and PFT measurements for each cluster. Entries are mean (SD) unless otherwise specified. The differences between the means of independent groups were analyzed by Welch’s ANOVA with the Games–Howell method for post hoc pairwise tests. The chi-square test was used to examine the relationship between two categorical variables.

	C0	C1	C2	C5	*p*
**Age (yrs.)**	54.41	48.96	40.89	49.91	0.005
	(15.55)	(14.43)	(12.03)	(14.36)	
**BMI (kg/cm^2^)**	23.74	25.14	22.29	23.65	0.038
	(3.19)	(2.69)	(3.37)	(3.03)	
**Height (cm)**	161.55	163.35	166.96	168.33	<0.001
	(8.40)	(7.44)	(8.32)	(7.61)	
**Weight (kg)**	62.16	67.43	62.65	67.25	0.142
	(10.81)	(11.12)	(13.24)	(10.96)	
**FVC (% pred)**	81	93.7	94.75	91	0.104
	(18.34)	(16.74)	(14.60)	(11.17)	
**FEV1 (% pred)**	82.58	98.48	101.18	94.76	0.064
	(24.40)	(17.38)	(13.86)	(18.15)	
**Gender (%)**	59.4/40.6	52.2/47.8	39.3/60.7	15.2/84.8	<0.001
**(Female/Male)**					
**Exposure (%)**	96.9/3.1	87.0/13.0	82.1/17.9	51.5/48.5	<0.001
**(Yes/No)**					
**Time of Exposure (hrs.)**	15,075.72	11,293.16	10,459.82	15,928.53	0.61
	(18,150.32)	(10,507.95)	(10,561.51)	(21,145.16)	
**PHMG or PGH (Count)**	17	14	13	9	0.65
**CMIT or MIT (Count)**	1	2	1	0	
**PHMG AND CMIT (Count)**	9	2	5	6	
**Other HDs (Count)**	2	1	3	2	

**Table 3:** Counts of HD-exposed and unexposed subjects in each cluster.

Exposure	No	Yes	Total
Cluster			
**C0**	1	31	32
**C1**	3	20	23
**C2**	5	23	28
**C3**	0	1	1
**C4**	0	4	4
**C5**	16	17	33

**Table 4:** Pair-wise contrasts for C0, C1, and C2, with C5 as a reference group.

	C0	C1	C2	C5
**Height**	--	--	-	
**BMI**		+		
**FVC % pred**				
**FEV_1_ % pred**				
**RV/TLC**	+	-	-	
**RV**	-	-	-	
**TLC**	---			
**LAA_RV_%**	-	--	--	
**fSAD%**	-	--	--	
**Tissue%**	+++	++		
**LAA_TLC_%**	--	--	-	
**F1**			+++	
**F2**	--			+++
**F3**	---	+++		
**F5**				+++

Note: “+” and “-” denote the relative magnitude of a given contrast being greater or smaller.

## Data Availability

This study was funded by the National Research Foundation of Korea and the Korean Ministry of Environment. Thus, any request for imaging data access shall be sent to Professor Chang Hyun Lee, Department of Radiology, Seoul National University, College of Medicine, Seoul, Korea (changhyun.lee@snu.ac.kr) as the point of contact for this project. The processed data presented in this study are openly available in FigShare at https://doi.org/10.6084/m9.figshare.21113251 (accessed on 5 August 2022).

## Supplementary Materials

The following supporting information can be downloaded at: https://www.mdpi.com/article/10.3390/ijerph191911894/s1, Figure S1. Training losses for CAE-FC models trained using different numbers of embeddings (pattern-clusters). Table S1. Post-hoc comparisons of the clinical and imaging-based variables between the clusters. The comparison pairs which were significantly different are shaded with gray. The differences between the means of independent groups were analyzed by Welch’s ANOVA with the Games-Howell method for post-hoc pairwise tests. Reference [35] is cited in the Supplementary Materials.

## Abbreviations

BMI	Body mass index
PFT	Pulmonary function test
EX	Expiration
IN	Inspiration
FEV_1_	Forced expiratory volume in one second
FVC	Forced vital capacity
HD	Humidifier disinfectant
HDLI	HD-associated lung injuries
CT	Computed tomography
EFA	Exploratory factor analysis
IRB	Institutional review boards
ROI	Regions of interest
CAE	Convolutional autoencoder
FC	Feature constructor
AWV%	Airway tree to lung volume ratio
RV/TLC	Residual volume to total lung capacity ratio
CFPD	Computational fluid and particle dynamics
LES	Large eddy simulation
F0	Factor 0
F1	Factor 1
F2	Factor 2
F3	Factor 3
F4	Factor 4
F5	Factor 5
C0	Cluster 0
C1	Cluster 1
C2	Cluster 2
C3	Cluster 3
C4	Cluster 4
C5	Cluster 5
*Variables below measured at different locations are denoted by {**Variable**}_{**Location**}.*	
**Variable**	
D_h_	Hydraulic luminal diameter
LAA_RV_%	Low attenuation area percentage at residual volume
LAA_TLC_%	Low attenuation area percentage at total lung capacity
fSAD%	Functional small airway disease percentage
*J*	Determinant of the Jacobian matrix
**Location**	
LUL	Left upper lobe
LLL	Left lower lobe
RUL	Right upper lobe
RML	Right middle lobe
RLL	Right lower lobe
Total	Total lung
sLUL	Sub-lobar subset airways at LUL
sLLL	Sub-lobar subset airways at LLL
sRUL	Sub-lobar subset airways at RUL
sRML	Sub-lobar subset airways at RML
sRLL	Sub-lobar subset airways at RLL
lLUL	Lobar airway at LUL
lLLL	Lobar airway at LLL
Lobar airway at RUL	Lobar airway at RUL
Lobar airway at RML	Lobar airway at RML
Lobar airway at RLL	Lobar airway at RLL
