# Supplementary material for: Characterizing Subjects Exposed to Humidifier Disinfectants Using Computed-Tomography-Based Latent Traits: A Deep Learning Approach
Source: Int J Environ Res Public Health. 2022 Sep 20;19(19):11894. doi: 10.3390/ijerph191911894 (PMC9565839; doi:10.3390/ijerph191911894)
Supplement: Supplementary file 1 [file ijerph-19-11894-s001.zip › ijerph-1878886-supplementary.pdf]

SUPPLEMENTARY MATERIALS

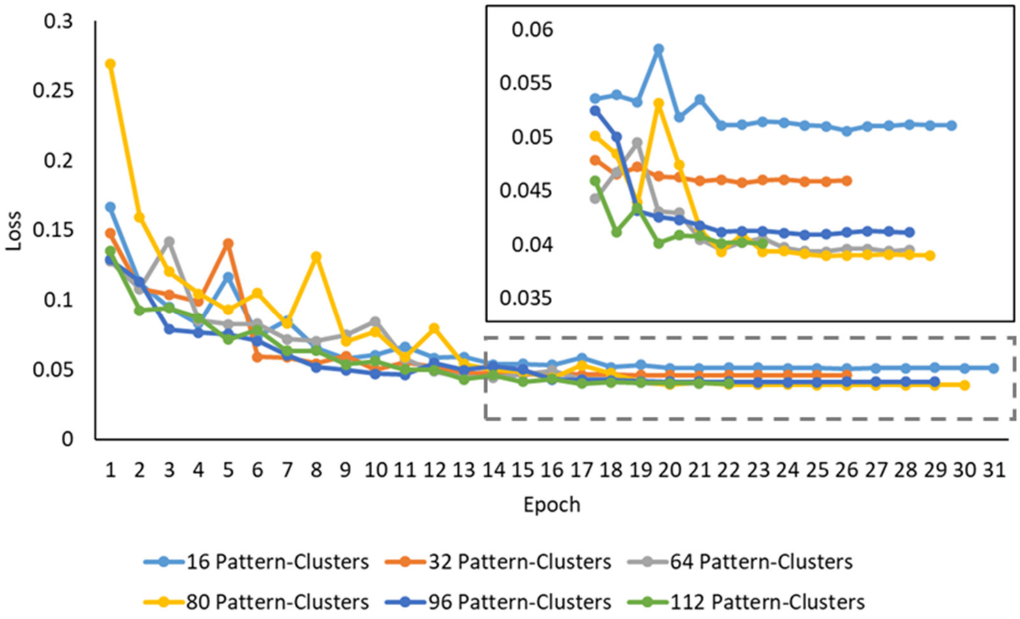

Figure S1. Training losses for CAE-FC models trained using different numbers of embeddings (pattern-clusters).

Steps of Exploratory Factor Analysis

Below description are reprinted/adapted with permission from the supplementary material of Ref. [1], 2021, Li et al.

The first step is factor extraction. We used principal axis factoring (PAF) [2] as our feature extraction method. It decomposes the correlation matrix of the observed variables to obtain the eigenvalues and eigenvectors, which represent the magnitude and directions of the new feature space, respectively. PAF assumes that the total variance of the observed variables is a combination of common variance and unique variance. Common variance is the variance that is shared among the observed variables while unique variance is the variance that is not shared in common (e.g.

noise). PAF is similar to principal component analysis (PCA), but it accounts for the unique variance of each variable while PCA does not.

The second step is factor rotation. The purpose of factor rotation is to obtain a simple structure for easier interpretation of the extracted factors. A simple structure means that each variable has high loadings on one factor only and each factor has high loadings for only some of the variables. Without rotation, the first factor will be the most general factor that most variables load onto and will explain the largest amount of variance, making it difficult to interpret. We used the OBLIMIN method, an oblique rotation method allowing correlations among factors, to rotate the extracted factors [2]. After factor rotation, we obtained a pattern matrix and a structure matrix. Coefficients of the pattern matrix reflect the unique factor loadings (similar to partial standardized regression coefficients), while the structure matrix contains correlations between factors and variables.

Finally, we calculated the factor scores (F_s) using **Equation (S1)** and **Equation (S2)** based on Thurstone's approach (3) for the future predictions of PFT measures or other clinical variables.

$$F_s = XW \quad \textbf{(S1)}$$

$$W = R^{-1}S = R^{-1}P\emptyset \quad \textbf{(S2)}$$

where X is the standardized pattern-histogram, W contains the regression weights, R is the correlation matrix of the pattern-histogram, S is the structure matrix, P is the pattern matrix, and $\emptyset$ is the correlation matrix of the factors. EFA was performed using R software (version 3.5.3) with the psych package (version 1.8.12).

Table S1. Post-hoc comparisons of the clinical and imaging-based variables between the clusters. The comparison pairs which were significantly different are shaded with gray. The differences between the means of independent groups were analyzed by Welch's ANOVA with the Games-Howell method for post-hoc pairwise tests.

Variable	Cluster (A)	Cluster (B)	mean(A)	mean(B)	p
Age	0	2	54.17	40.65	0.003
Age	2	5	40.65	49.91	0.033
Height	0	2	159.74	166.89	0.011
Height	0	5	159.74	168.33	0.001
BMI	1	2	25.15	22.48	0.016
RV/TLC	0	1	0.77	0.42	0.001
RV/TLC	0	2	0.77	0.39	0.001
RV/TLC	0	5	0.77	0.55	0.012
RV/TLC	1	5	0.42	0.55	0.001
RV/TLC	2	5	0.39	0.55	0.001
AirT%_Total	0	1	16.43	3.04	0.001
AirT%_Total	1	2	3.04	9.34	0.001
AirT%_Total	1	5	3.04	21.21	0.001
AirT%_Total	2	5	9.34	21.21	0.001
fSAD%_Total	0	1	10.52	0.68	0.005
fSAD%_Total	1	2	0.68	3.67	0.001
fSAD%_Total	1	5	0.68	11.54	0.001
fSAD%_Total	2	5	3.67	11.54	0.001
Tissue%_Total	0	1	16.98	13.63	0.021
Tissue%_Total	0	2	16.98	9.60	0.001
Tissue%_Total	0	5	16.98	10.51	0.001
Tissue%_Total	1	2	13.63	9.60	0.001
Tissue%_Total	1	5	13.63	10.51	0.001
Tissue%_Total	2	5	9.60	10.51	0.014
Emph%_Total	0	5	1.33	3.46	0.023
Emph%_Total	1	2	1.21	3.16	0.006
Emph%_Total	1	5	1.21	3.46	0.002
Age	0	1	54.17	48.09	0.490
Age	0	5	54.17	49.91	0.679
Age	1	2	48.09	40.65	0.202
Age	1	5	48.09	49.91	0.900
Height	0	1	159.74	163.36	0.378
Height	1	2	163.36	166.89	0.434
Height	1	5	163.36	168.33	0.087
Height	2	5	166.89	168.33	0.900
BMI	0	1	24.05	25.15	0.546

BMI	0	2	24.05	22.48	0.306
BMI	0	5	24.05	23.65	0.900
BMI	1	5	25.15	23.65	0.233
BMI	2	5	22.48	23.65	0.513
RV/TLC	1	2	0.42	0.39	0.481
AirT%_Total	0	2	16.43	9.34	0.176
AirT%_Total	0	5	16.43	21.21	0.547
fSAD%_Total	0	2	10.52	3.67	0.100
fSAD%_Total	0	5	10.52	11.54	0.900
Emph%_Total	0	1	1.33	1.21	0.900
Emph%_Total	0	2	1.33	3.16	0.057
Emph%_Total	2	5	3.16	3.46	0.900

Reference

1. Li, F.; Choi, J.; Zou, C.; Jr., J.D.N.; Comellas, A.P.; Lee, C.H.; Ko, H.; Barr, R.G.; Bleecker, E.R.; Cooper, C.B.; et al. Latent traits of lung tissue patterns in former smokers derived by dual channel deep learning in computed tomography images. Sci. Rep. 2021, 11, 4916. https://doi.org/10.1038/s41598-021-84547-5. (accessed on 1 March 2021)
2. Dunlap, J.W.; Thurstone, L.L. The Vectors of the Mind. *Am. J. Psychol.* **1937**, *49*, 329.
3. Costello, A.B.; Osborne, J.W. Best practices in exploratory factor analysis: Four recommendations for getting the most from your analysis. *Pract. Assess. Res. Eval.* **2005**, *10*, 7.
